# Proteomic analysis of sera of asymptomatic, early-stage patients with Wilson's disease

**DOI:** 10.1002/prca.200800057

**Published:** 2009-10

**Authors:** Jung-Young Park, Joo Hee Mun, Beom Hee Lee, Sun Hee Heo, Gu-Hwan Kim, Han-Wook Yoo

**Affiliations:** 1 Genome Research Center for Birth Defects and Genetic Diseases, Asan Institute for Life SciencesSeoul, Korea; 2 Department of Pediatrics, Asan Medical Center, University of Ulsan College of MedicineSeoul, Korea

**Keywords:** 2-DE, Wilson's disease

## Abstract

Wilson's disease (WD) is characterized by excessive accumulation of intracellular copper in liver and extrahepatic tissues, leading to significant oxidative stress and tissue damage. To date, several diagnostic biomarkers for WD such as serum ceruloplasmin, serum or urine copper levels and copper content in liver have been identified. However, these biomarkers may not be convincing for the diagnosis in some WD patients. To identify additional novel diagnostic biomarkers, we compared the serum protein profiles of asymptomatic childhood WD patients (*n*=20), without neurologic manifestation or liver cirrhosis, with normal controls (*n*=13). Fourteen spots, five up-regulated and nine down-regulated (>2-fold), were differentially expressed in WD patients in comparison to normal control on 2-DE. Among them, three spots were down-regulated in both male and female WD. MS/MS analysis revealed that the three spots were complement component C3, complement factor B and alpha-2 macroglobulin. By comparative proteome analysis, complement component C3, complement factor B and alpha-2 macroglobulin, which are related to oxidative stress and inflammation, turned out to be good candidates for novel diagnostic biomarkers for early stages of WD.

## 1 Introduction

Copper, an essential trace element in living organisms, acts as a cofactor for several enzymes due to its biologically suitable redox potential. However, excess copper leads to random oxidation of biomolecules with subsequent toxic effects 1. Wilson's disease (WD) is an autosomal recessive inherited copper metabolism disorder caused by mutation of the *ATP7B* gene 2,3. The incidence of WD is estimated as one in 30 000 in most populations 4. Alterations in the ATP7B protein cause impaired copper efflux from the liver into bile and decreased incorporation of copper into ceruloplasmin (CP), leading to its accumulation in the liver 5. Manifestation of WD can be variable. It can be incidentally detected in asymptomatic patients with abnormal liver function alone, whereas symptomatic, early-stage patients can be found with chronic liver disease, liver cirrhosis or neurological impairment. The early diagnosis of WD is important because it can be lethal when left untreated. Patients with WD show decreased serum CP, increased urinary excretion of copper, elevated hepatic copper concentration and Kayser–Fleischer rings on cornea 6,7. Although serum CP, serum/urine copper levels and copper content in liver tissue are considered as diagnostic biomarkers of WD, 5–40% of the patients with WD display normal CP levels, and serum/urine copper levels may be equivocal 8,9. In addition, invasive study is often required to evaluate copper content in liver. Therefore, diagnosis can be indeterminate, especially in asymptomatic, early-stage patients.

This study was undertaken to find novel biomarkers of asymptomatic, early-stage WD. Using comparative proteome analyses, including multiple affinity removal columns (MARC), 2-DE and MALDI-TOF, we identified the differently expressed proteins and we discussed their functional roles in asymptomatic WD patients.

## 2 Materials and methods

### 2.1 Serum sample preparation

Serum samples were collected from 20 asymptomatic childhood WD patients, 12 males and 8 females, without neurologic manifestation or liver cirrhosis. All of them were detected by abnormal serum liver enzyme levels, which were incidentally found during health screening or preoperative examinations for minor surgical procedures not related to WD, such as tonsillectomy. WD was diagnosed based on low serum CP levels (<15 mg/dL) and increased urinary copper excretion (>100 μg/day). Thirteen age- and sex-matched individuals, six males and seven females, with normal liver functions and CP levels were classified as normal group. WD patients with same gender were randomly divided into two groups, male WD patients (mWD1/2) and female WD patients (fWD1/2), to give each group the same number of patients (Table 1). Samples from normal group and mWD1/2 and fWD1/2 were split into 300 μL aliquots, frozen and stored at −80°C. The total protein content was determined using the Bradford method, according to the manufacturer's instructions (Bio-Rad Laboratories, Hercules, CA, USA). Written informed consent was obtained from each individual, and the research protocol approved by the Institutional Review Board (IRB) of Asan Medical Center, University of Ulsan College of Medicine (Seoul, Korea).

**Table 1:** Clinical data on control subjects and WD patients

Sex	Group	Number	Age mean (range)	CP (mg/dL) mean (±SD)	Copper serum (ug/dL) mean (±SD)	AST(IU/L)a) mean (±SD)	ALT(IU/L)b) mean (±SD)
			mean (range)	mean (±SD)	mean (±SD)	mean (±SD)	mean (±SD)
Male	Normal	6	9 (5–12)	33 (±6.5)	115 (±17.5)	35 (±10)	25 (±10)
	mWD1c)	6	9 (5–12)	2.2 (±0.7)	13.4 (±7.9)	279 (±228)	361 (±241)
	mWD2	6	9 (6–12)	4.1 (±2.0)	18.8 (±9.9)	221 (±193)	260 (±224)
Female	Normal	7	7 (5–12)	32 (±5)	105 (±17.5)	27.5 (±6.3)	17.5 (±8.8)
	fWD1d)	4	8 (5–12)	3.2 (±0.7)	22.6 (±17.6)	143 (±84)	242 (±187)
	fWD2	4	8 (6–10)	2.7 (±0.4)	18.4 (±8.6)	134 (±67)	218 (±102)

a) Aspartate aminotransferase: GOT.

b) Alanine aminotransferase: GPT.

c) Male Wilson's disease group.

d) Female Wilson's disease group.

### 2.2 Depletion of major abundant proteins using MARC

Serum depletion was performed as described previously 10. The six most abundant proteins in each serum sample were depleted using 4.6×100 mm MARC (Agilent, Wilmington, DE, USA) with a binding capacity of 20 μL of human serum. Chromatographic separation was performed using a mobile phase reagent kit, according to the standard LC protocol provided by the manufacturer. Serum samples were diluted fivefold with buffer A containing protease inhibitors (COMPLETE™, Roche), and filtered through 0.22 μm spin filters at 12 000 rpm for 1 min at room temperature. Flow-through MARC fractions were pooled and precipitated with a precooled solution of 10% trichloroacetic acid for 1 h at −20°C. Each precipitate was washed with ice-cold acetone, and redissolved in sample lysis buffer (7 M urea, 2 M thiourea, 100 mM DTT, 4.5% CHAPS and 40 mM Tris).

### 2.3 2-DE

MARC-treated samples (800 μg) were mixed with rehydration buffer (8 M urea, 2% CHAPS, 65 mM DTT, 0.5% IPG buffer), according to the manufacturer's instructions, to a final volume of 340 μL, and applied onto immobilized pH 3–10NL strips (Amersham Biosciences, Uppsala, Sweden). Isoelectric focusing was performed at 80 000 Vh. Strips were applied to 12.5% polyacrylamide gels and electrophoresed until the dye reached end of the gel. After protein fixation for 1 h, gels were stained with Coomassie Brilliant Blue G250 for 24 h, destained with H_2_O, scanned in a Umax power Look 1100 (Umax data system) and converted into electronic files, which were analyzed with the Image Master Platinum 5.0 image analysis program (Amersham Biosciences). Spot detection parameters were adjusted using a smooth by Algorithm. The number of spots was automatically determined and matched. The normalized volume was quantified as percentage volume (%*v*), where %*v*=spot volume/Σvolumes of all spots resolved in the gel. Variations greater than twofold were regarded as unique, representing differentially expressed proteins.

### 2.4 Identification of proteins using MS/MS

Protein spots were excised from CBB-stained gels, and digested with trypsin (Promega, Madison, WI, USA). Tryptic peptides were concentrated on POROS R2 and oligo R3 columns (Applied Biosystems, Foster City, CA, USA). After washing each column with 70 and 100% ACN and 50 mM ammonium bicarbonate, samples were applied to the R2 and R3 columns, and eluted with CHCA (Sigma, St. Louis, MO) dissolved in 70% ACN and 0.1% TFA before MALDI-TOF analysis 11. Mass spectra were acquired on a 4700 Proteomics Analyzer (Applied Biosystems) operated in the MS and MS/MS modes. The mass spectrometer was set to acquire positive ion MS survey scans over the mass range of 700–4000 Da. The data were processed to generate a list of precursor ions for interrogation by MS/MS. Each raw spectrum was opened in Data Explorer software and less than 40 top mass peaks were selected by *S*/*N* threshold setting and then treated with advanced base-line correction. The spectrum was calibrated with more than two peptides resulting from trypsin autolysis (*m*/*z* 842.5100, 2211.1046). Peak list of monoisotopic masses was searched using the following parameters: trypsin as enzyme, one possible missed cleavage, peptide tolerance of 0.5 Da and mass tolerance of 100 ppm. Peptide mass fingerprinting was performed using the MASCOT search engine (http://matrixscience.com/) included in GPS Explorer software and ProFound program (http://prowl.rockefeller.edu/). Mass spectra were used for manual *de novo* sequencing, and sequences annotated with Data Explorer software (Applied Biosystems). Peptide matching and protein searches were performed using Swiss-Prot and NCBI databases (ver.20061110, 4111659 sequences; 1417848065).

### 2.5 Western blot analysis

Serum proteins (30 μg) were subjected to electrophoresis on 10% SDS-polyacrylamide gels, and transferred to PVDF membranes (Bio-Rad). After overnight blocking with blocking buffer (TBS, 5% skimmed milk, 0.1% Tween 20), membranes were incubated with polyclonal antibodies against alpha-2 macroglobulin (α2M), complement component C3 (C3), and complement factor B (FB) (Santa Cruz Biotechnology, Santa Cruz, CA, USA), followed by HRP-labeled secondary antibody. Blots were visualized using the SuperSignal West Pico Chemiluminescent Substrate (PIERCE, UK) detection system, according to the manufacturer's instructions. An antibody against glyceraldehyde-3-phosphate dehydrogenase (GAPDH) was employed as the internal control.

### 2.6 Statistical analysis

All statistical analyses were performed using SPSS 12.0 software. Student's *t*-test was applied to compare differences between control subjects and WD patients.

## 3 Results

**Figure 1:**
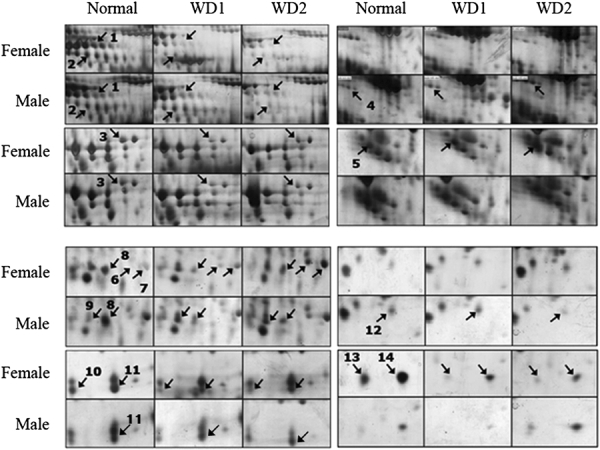
Partial 2-DE comparison images of normal subjects and asymptomatic WD patients. Differentially expressed proteins displaying >2-fold difference in levels are indicated with numbered arrows.

**Figure 2:**
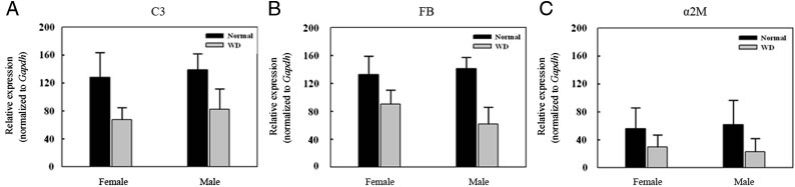
Differential expression patterns of proteins between normal subjects and asymptomatic WD patients. C3 (A), FB (B) and α2M (C) were less abundant in WD patients, as established with MS and MS/MS, as well as western blotting using polyclonal antibodies. Following normalization relative to GAPDH, expression of C3, FB and α2M was significantly lower in asymptomatic WD patients than in normal subjects. ^*^p<0.005 and ^**^p<0.001 with Student's *t*-test.

Serum protein profiles of mWD1/2, fWD1/2 and normal individuals were analyzed using 2-DE, visualized by staining with Coomassie Brilliant Blue G-250, and compared using the Image Master Platinum 5.0 program (data not shown). Comparing serum protein profiles between mWD1/2 and fWD1/2 and normal control, five spots were up-regulated (spots 4–7 and 9) and nine down-regulated (spots 1–3, 8, and 10–14) by at least twofold in depleted sera of asymptomatic WD regard1ess of sex (Fig. 1). The selected spots were re-analyzed using MALDI-TOF MS and subsequently confirmed by MS/MS (Table 2). Finally, spots 1, 2, 3, 8 and 11 were confirmed to be down-regulated in both male and female WD patients (Fig. 1 and Table 2). Spots 1 and 3, spot 2 and spot 11 were FB, C3 and α2M precursors, respectively. Western blot analysis for C3, FB and α2M also showed decreased expressions in WD patients (Fig. 2 and Supporting Information Fig. S1). Following normalization in relation to GAPDH expression, C3, FB and α2M levels were still decreased significantly in the sera of WD patients in comparison with those of normal subjects (Fig. 2). α2M expression was decreased to 2.3-fold in male and 1.8-fold in female WD patients (*p*<0.001), C3 to 1.8-fold in male and 1.9 in female (*p*<0.005) and FB to 2.4-fold in male and 1.6-fold in female (*p*<0.005).

**Table 2:** Differentially expressed proteins in sera of normal subjects and asymptomatic WD patients, identified using MALDI-TOF MS and MS/MS

Spot no.	Protein name	Up/down-regulated female/malea)	Accession no.		MALDI-TOF MS			MALDI-TOF MS/MSb)	
			NCBInr	Swiss-Prot	Score	Decision DB (MASCOTc)/ProFoundd))	Matched peptides (%)	MASCOT score	Matched peptides (%)
1	Complement factor B, preproprotein	−5.5/−3.1	gi∣4502397	Q53F89	103	MASCOT	11/25 (44%)	242	4/43 (9%)
2	Alpha-2-macroglobulin precursor	−4.3/−2.7	gi∣112911	P01023	1531.3e-03	MASCOTProFound	17/25 (68%)	469	4/53 (8%)
3	Complement factor B preproprotein	−2.4/−2.2	gi∣14124934	Q53F89	82	MASCOT	25/210 (12%)	141	3/76 (4%)
4	Hemoglobin Gower 2 epsilon	–/+3.4	gi∣223114	P02100	71	MASCOT	6/40 (15%)		
5	Nesprin-1 beta 2	+4.4/–	gi∣370178	Q8NF91	65	MASCOT	42/135 (31%)		
6	Immunoglobulin heavy chain variable region	+3.0/–	gi∣18307244	P01764	34	MASCOT	5/128 (4%)		
7	Ig heavy chain V region (clone P1–53)	+3.3/–	gi∣484705	P23083	66	MASCOT	5/25 (20%)		
8	Isocitrate dehydrogenase 3 (NAD+) alpha	−2.1/−2.8	gi∣5031777	P50213				15	1/49 (2%)
9	Immunoglobulin heavy chain variable region	–/+2.5	gi∣112701283	P01764	60	MASCOT	5/42 (12%)		
10	Cytokeratin 8	−2.0/–	gi∣30313	P05787				63	1/47 (2%)
11	Complement component C3 precursor	−2.3/−2.1	gi∣4557385	P01024	119	MASCOT	17/115 (15%)	302	5/72 (7%)
12	Thiol-specific antioxidant protein (PRX2)	–/−2.0	gi∣438069	P32119	87	MASCOT	13/155 (8%)	320	6/71 (9%)
13	Smad ubiquitination regulatory factor 1	−6.9/–	gi∣10047327	Q9HCE7				24	1/44 (2%)
14	Haptoglobin precursor	−3.8/–	gi∣67586	P00738				24	1/25 (4%)

a) Fold changes in values obtained from image analysis of spots between control subjects and patients.

b) Individual ions scores >35 indicate identity or extensive homology (*p*<0.05). Ions score is –10 log(*p*), where *p* is the probability that the observed match is a random event.

c) Protein scores greater than 64 are significant (*p*<0.05) for MS data.

d) Expectation value scoring was used, which indicates the quality or significance of the match.

## 4 Discussion

In this study, three proteins, C3, FB and α2MG, were differentially expressed in asymptomatic WD patients. These proteins are known to be related with several functions including inflammation, regulation of the cell cycle and protection of cells against oxidative stress. C3 plays a central role in activation of the classical and alternative complement activation pathways 12, while FB is a crucial component that regulates alternative complement pathway 13,14. These proteins are synthesized primarily in the liver, which is induced during acute inflammatory processes. Down-regulations of C3 and FB in WD imply reduced activity of complement activation pathway in WD patients, which may be caused by excessive consumption and/or decreased synthesis due to hepatic copper toxicity.

α2M, a universal protease inhibitor and cytokine transporter, is an evolutionarily conserved component of the innate immune system 15,16. Moreover, α2M is a large molecular-sized plasma protein produced by hepatocytes 17 that is responsible for the inhibition and clearance of proteases secreted by endogenous and exogenous pathogens from the circulation 16. Severe hepatocellular damage and chronic liver disease lead to reduced production of the α2M protein 18. However, the WD patients enrolled in this study did not have a severe hepatic damage but elevated serum liver enzyme levels, which might lead to reduced production. Therefore, reduced expression of α2M in asymptomatic WD patients may indicate blunted acute phase response to early stage of hepatitis induced by copper toxicity.

Besides three proteins, which were down-regulated, several proteins were differentially expressed in WD patients to a lesser degree (Table 2). Nesprin, which was up-regulated only in female WD, plays several fundamental roles including maintenance of structural organization, regulation of the cell cycle and maintaining/targeting protein complexes common to the nuclear envelope and sarcoplasmic reticulum, particularly in differentiated muscle cells 19. Elevation of nesprin-1 beta2 may reflect damage to the cell membrane and cytoskeleton resulting from intracellular copper accumulation. Peroxiredoxin 2 isoform b with down-regulation in male WD is an intracellular antioxidant protein that protects cells from oxidative stress through redox regulation 20. A reduction in the amount of peroxiredoxin 2 isoform b may therefore suggest increased cell susceptibility to oxidative damage induced by intracellular copper accumulation 21. Smad proteins mediate signaling by members of the transforming growth factor-beta superfamily 22. Smurf1 is a member of the Hect domain family of E3 ubiquitin ligases, which interacts with the bone morphogenic protein (BMP) signaling proteins, Smad1 and 5, the bone-specific transcription factor, Runx2/Cbfa1, and type I BMP receptors mediating their degradation 23. Additionally, Smurf1 possibly plays an important role in osteoblast differentiation 24. The relationship between decreased expression of Smurf1 in female WD and the pathogenetic effect on WD remains elusive, which is needed to be investigated. Haptoglobin, a liver glycoprotein, is primarily secreted by liver cells in response to a variety of stimuli 25. This hemoglobin-binding serum protein participates in protection against heme-driven oxidative stress 26. Low haptoglobin levels are reported in various clinical conditions, including tumor metastasis, severe sepsis, hemolytic anemia and chronic liver disease 27,28. Moreover, haptoglobin has been reported as a potential serum biomarker of ovarian cancers, 29,30. In our analysis, haptoglobin precursor was down-regulated in female WD (Table 2). In order to verify this result, we performed Western blot analysis with the whole sera of WD patients including male and female. The proportion of haptogloblin was decreased in both male and female WD compared with normal. However, haptogloblin of female WD showed a little more down-regulated pattern than male WD (Supporting Information Fig. S2). Profound depletion of haptoglobin precursor may imply the presence of asymptomatic hemolysis caused by copper toxicity to red blood cells as well as reduced synthesis from the damaged liver, but the reason for gender difference is yet to be known.

In conclusion, our experiments indicate that C3, FB and α2M are down-regulated in asymptomatic WD patients implying impaired cellular response to inflammatory stimulus, which might be caused by intracellular copper accumulation in hepatic tissue. These proteins can be candidates for novel diagnostic biomarkers for WD, particularly in the asymptomatic stage.
