# Supplementary material for: Proteomic analysis of sera of asymptomatic, early-stage patients with Wilson's disease
Source: Proteomics Clin Appl. 2009 Oct;3(10):1185–90. doi: 10.1002/prca.200800057 (PMC2883077; doi:10.1002/prca.200800057)
Supplement: Supplementary file 1 [file prca0003-1185-SD1.pdf]

PROTEOMICS

Supporting Information
for Proteomics Clin. Appl.

DOI 10.1002/prca.200800057

Jung-Young Park, Joo Hee Mun, Beom Hee Lee, Sun Hee Heo,

Gu-Hwan Kim and Han-Wook Yoo

Proteomic analysis of sera of asymptomatic, early-stage patients with Wilson's disease

© 2009 WILEY-VCH Verlag GmbH & Co. KGaA, Weinheim

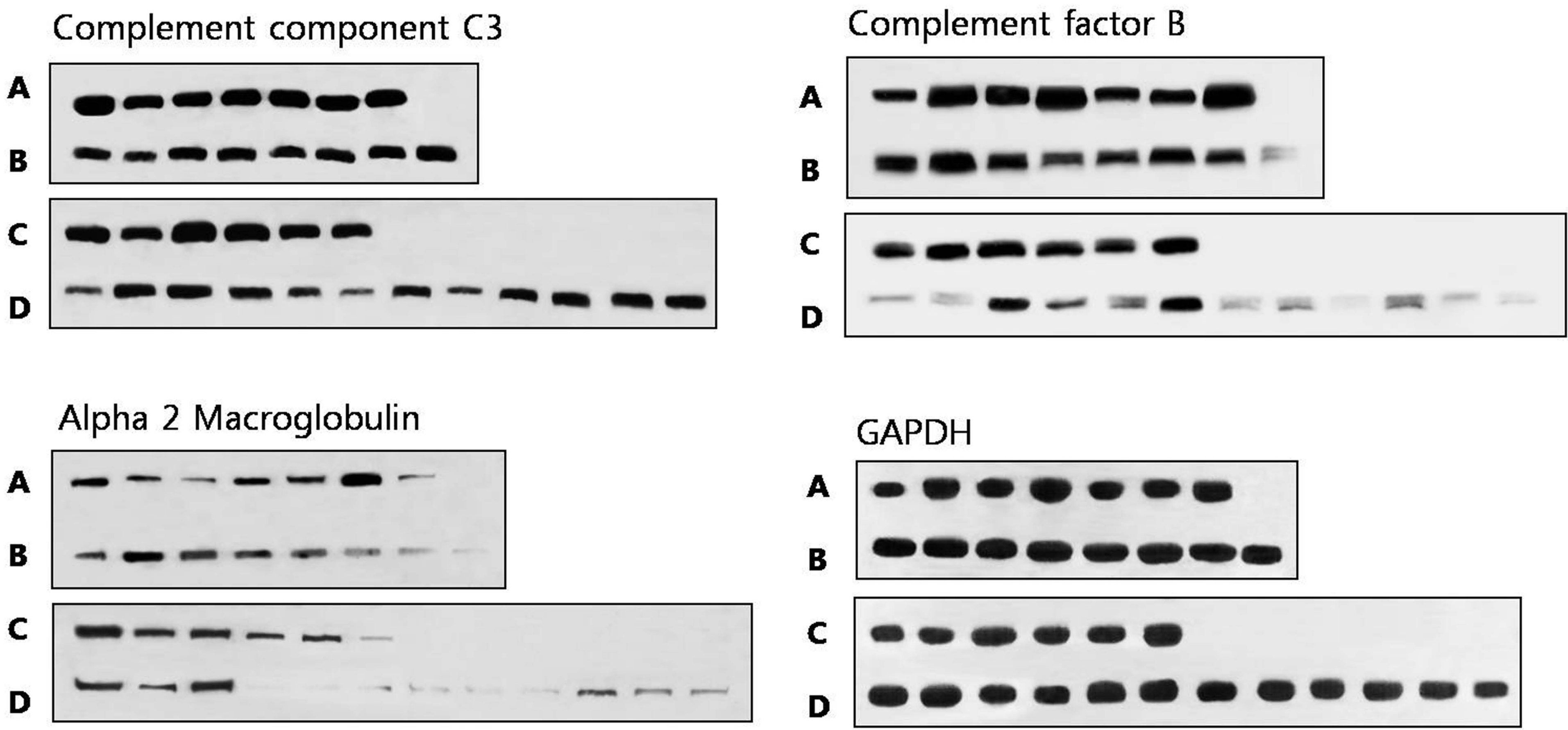

Complement component C3
A
B
C
D
Complement factor B
A
B
C
D
Alpha 2 Macroglobulin
A
B
C
D
GAPDH
A
B
C
D

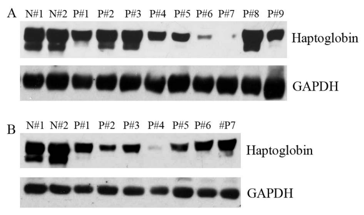
A
N#1
N#2
P#1
P#2
P#3
P#4
P#5
P#6
P#7
P#8
P#9
Haptoglobin
GAPDH
B
N#1
N#2
P#1
P#2
P#3
P#4
P#5
P#6
#P7
Haptoglobin
GAPDH
